# Comparative Effects of n-3, n-6 and n-9 Unsaturated Fatty Acid-Rich Diet Consumption on Lupus Nephritis, Autoantibody Production and CD4^+^ T Cell-Related Gene Responses in the Autoimmune NZBWF1 Mouse

**DOI:** 10.1371/journal.pone.0100255

**Published:** 2014-06-19

**Authors:** James J. Pestka, Laura L. Vines, Melissa A. Bates, Kaiyu He, Ingeborg Langohr

**Affiliations:** 1 Department of Food Science and Human Nutrition, Diagnostic Center for Population and Animal Health, Michigan State University, East Lansing, Michigan, United States of America; 2 Center for Integrative Toxicology, Diagnostic Center for Population and Animal Health, Michigan State University, East Lansing, Michigan, United States of America; 3 Department of Microbiology and Molecular Genetics, Diagnostic Center for Population and Animal Health, Michigan State University, East Lansing, Michigan, United States of America; 4 Division of Anatomic Pathology, Diagnostic Center for Population and Animal Health, Michigan State University, East Lansing, Michigan, United States of America; Max Delbrueck Center for Molecular Medicine, Germany

## Abstract

Mortality from systemic lupus erythematosus (SLE), a prototypical autoimmune disease, correlates with the onset and severity of kidney glomerulonephritis. There are both preclinical and clinical evidence that SLE patients may benefit from consumption of n-3 polyunsaturated fatty acids (PUFA) found in fish oil, but the mechanisms remain unclear. Here we employed the NZBWF1 SLE mouse model to compare the effects of dietary lipids on the onset and severity of autoimmune glomerulonephritis after consuming: 1) n-3 PUFA-rich diet containing docosahexaenoic acid-enriched fish oil (DFO), 2) n-6 PUFA-rich Western-type diet containing corn oil (CRN) or 3) n-9 monounsaturated fatty acid (MUFA)-rich Mediterranean-type diet containing high oleic safflower oil (HOS). Elevated plasma autoantibodies, proteinuria and glomerulonephritis were evident in mice fed either the n-6 PUFA or n-9 MUFA diets, however, all three endpoints were markedly attenuated in mice that consumed the n-3 PUFA diet until 34 wk of age. A focused PCR array was used to relate these findings to the expression of 84 genes associated with CD4^+^ T cell function in the spleen and kidney both prior to and after the onset of the autoimmune nephritis. n-3 PUFA suppression of autoimmunity in NZBWF1 mice was found to co-occur with a generalized downregulation of CD4^+^ T cell-related genes in kidney and/or spleen at wk 34. These genes were associated with the inflammatory response, antigen presentation, T cell activation, B cell activation/differentiation and leukocyte recruitment. Quantitative RT-PCR of representative affected genes confirmed that n-3 PUFA consumption was associated with reduced expression of CD80, CTLA-4, IL-10, IL-18, CCL-5, CXCR3, IL-6, TNF-α and osteopontin mRNAs in kidney and/or spleens as compared to mice fed n-6 PUFA or n-9 MUFA diets. Remarkably, many of the genes identified in this study are currently under consideration as biomarkers and/or biotherapeutic targets for SLE and other autoimmune diseases.

## Introduction

Systemic lupus erythematosus (SLE), a debilitating chronic autoimmune disease affecting approximately 1 in 1000 persons in the U.S., has a complex etiology that involves genetic, environmental and nutritional interactions [1]. Critical events in the initiation of SLE include the impaired clearance of apoptotic cells by macrophages and aberrant presentation of self-antigens to T and B cells. This results in formation of autoantibody-autoantigen complexes and their subsequent deposition in the kidney and other tissues [2]. Collectively, these changes elicit cytokine/chemokine production, complement activation and infiltration with monocyte/macrophages, CD4^+^ T cells, CD8+ T cells, B cells and plasma cells that together evoke irreparable tissue damage [3]. CD4^+^ T cell activation is a hallmark of SLE and has been previously reported in autoimmune-prone mice [4]. CD4^+^ T cells compromise the majority of infiltrating cells in the kidneys of patients with active lupus nephritis and urinary concentrations of CD4^+^ T cells are correlated to severity of lupus nephritis [5]. Importantly, SLE mortality correlates with the development of autoimmune glomerulonephritis [6]. Because many SLE patients have untoward side effects from or are unresponsive to conventional drugs and biological therapeutics, they often seek complementary or alternative therapy options that include diet modification and use of nutritional supplements [7], [8]. Consumption of fish oil is one such approach that has potential to prevent and/or ameliorate SLE and other types of autoimmune glomerulonephritis [9].

Since humans and other mammals require but do not synthesize polyunsaturated fatty acids (PUFAs), it is essential that they consume these in their diet [10]. Linoleic acid (C18∶2n-6), is the major PUFA found in food oils derived from plants (e.g. corn and soybean) that are extensively used in Western diets. Following consumption and metabolism, linoleic acid elongates and desaturates to yield arachidonic acid (C20∶4n-6; AA). The Δ15- desaturase found in plants converts linoleic acid to α-linolenic acid which can be elongated to eicosapentaenoic acid (c20∶5n-3) (EPA) and docosahexaenoic acid (22∶6n-3) (DHA). While these latter two conversions occur slowly in mammals, they are readily carried out by marine algae. Fish consuming these algae readily incorporate EPA and DHA into their tissue, making fish and fish oils a source of preformed long chain n-3 PUFAs for human foods and dietary supplements. A CDC-NHIS survey found that nearly 30 million individuals of U.S. adults consume fish oil supplements because of perceived health benefits [11].

n-3 PUFAs suppress proinflammatory cytokine production, lymphocyte proliferation, cytotoxic T cell activity, natural killer cell activity, macrophage-mediated cytotoxicity, neutrophil/monocyte chemotaxis, MHCII expression and antigen presentation [10]. The potential of n-3 PUFAs to specifically delay, prevent and ameliorate SLE has been investigated extensively in mouse models. The female New Zealand Black White (F1) mouse (NZBWF1), a widely used model that closely resembles human SLE, spontaneously develops the characteristics of lupus nephritis between approximately 30 to 40 wk of age resulting in a drastically shortened lifespan [12]. Early studies with NZBWF1 mice demonstrated that initiating feeding of menhaden oil, DHA ethyl ester or EPA ethyl ester early in life markedly reduced severity and incidence of renal disease as well as extended lifespan compared to mice fed beef tallow [13], [14], [15]. These findings coincided with reductions of anti-ds-DNA and circulating immune complexes, biomarkers positively correlated with SLE disease activity. Elegant studies from the Fernandes laboratory have related the delayed onset and decreased severity of renal disease exhibited in fish oil-fed NZBWF1 mice to reduced IL-1β, TNF-α, TGFβ1, ICAM-1 and fibronectin expression and increased expression of antioxidant enzymes [16], [17], [18], [19], [20]. The ameliorative effects of n-3 PUFAs have been similarly replicated in two other murine SLE models, BXSB/MpJ and MRL-1pr/1pr, as evidenced by decreased proteinuria and glomerular injury as well as increased lifespan [14], [21], [22]. Effects of fish oil in MRL-1pr/1pr mice were linked to altered eicosanoid metabolism as well as decreased plasma IL-6, IL-10, IL-12 and TNF-α [23], [24], [25].

There is clinical evidence that consumption of fish oil benefits SLE patients. In a prospective double blind crossover study, 27 SLE patients were given either 20 g of fish oil daily or 20 g of olive oil for 12 wk as part of a standardized isoenergetic low fat diet [26]. When outcome measures were assessed, 14 out of 17 compliant patients who received fish oil showed benefits when evaluated for generalized lupus disease criteria. Wright and coworkers [27] conducted a 24 wk double-blind placebo-controlled parallel trial in which the effects of daily consumption of 3 g of n-3 PUFA (1.2 g DHA plus 1.8 g EPA) by SLE patients for 24 wk were compared to SLE patients consuming an olive oil placebo. There were significant improvements in systemic lupus scores and disease activity for SLE in those consuming n-3 PUFAs. It must be noted that, in contrast to the aforementioned studies, two other clinical trials reported that n-3 PUFAs were not effective in treating lupus nephritis [28], [29].

Many questions remain regarding the use of n-3 PUFA-containing oils to prevent or counter autoimmune responses in SLE and other autoimmune diseases concerning their efficacy relative to comparative effects of other unsaturated fatty acids, molecular mechanisms, cellular targets, potential biomarkers of effect and requisite dosages. In this investigation, we used the NZBWF1 SLE model to compare the effects on of autoimmunity onset as reflected by autoantibody production and glomerulonephritis in animals consuming: 1) an n-3 PUFA-rich diet containing docosahexaenoic acid enriched fish oil (DFO), 2) an n-6 PUFA-rich Western-style diet containing corn oil (CRN) or 3) an n-9 monounsaturated fatty acid (MUFA)-rich Mediterranean-style diet containing high oleic safflower oil (HOS). In addition, a focused PCR array was used to relate these findings to the expression of genes associated with CD4^+^ T cell function in spleen and kidney both prior to and after the onset of the autoimmune nephritis. Finally, the downregulation of selected genes from the array was confirmed by quantitative RT-PCR. Marked autoantibody and nephritic responses were observed to the same extent in mice fed either n-6 PUFA or n-9 MUFA diets, however, these effects were remarkably suppressed in mice consuming the n-3 PUFA diet. Notably, this suppression was concurrent with generalized downregulation of CD4**^+^** T cell-related gene expression of in kidney and/or spleen. Many of the genes identified in this study are under consideration as potential biomarkers and/or therapeutic targets for human SLE.

## Materials and Methods

### Animals

All animal protocols were reviewed and approved by Michigan State University’s Institutional Animal Care and Use Committee. Three-week-old female NZBWF1/J mice were purchased from Jackson Laboratory (Bar Harbor, ME) and provided food and water *ad lib*. Mice were housed 4 per cage under specific pathogen-free conditions using a HEPA filtered Innorack IVC cages (Innovive Inc., San Diego, CA).

### Diets

Standard purified isocaloric AIN-93G diets [30] (Dyets, Inc., Bethlehem, PA) containing 70 g total oil per kg were prepared (Table 1). Experimental diets were modified to contain 60 g/kg diet of oils from three different sources. DHA ethyl ester-enriched fish oil (DFO) from Ocean Nutrition Canada (Dartmouth, Nova Scotia) containing 540 g/kg DHA and 50 g/kg EPA was used for the n-3 PUFA- rich diet. Corn oil (CRN) from Dyets containing 612 g/kg linoleic acid (n-6) and 26 g/kg of oleic acid (n-9) was employed as a source of n-6 PUFA. High-oleic safflower oil (HOS) from Hain Celestial Group (Boulder, CO) containing 750 g/kg oleic acid and 140 g/kg linoleic acid was utilized as the n-9 MUFA source. To provide basal essential fatty acids, all diets were supplemented with corn oil (10 g/kg diet).

**Table 1:** Composition of experimental diets.

	Experimental Diet		
Ingredients^a^	CRN	HOS	DFO
	g/kg	g/kg	g/kg
Casein	200.00	200.00	200.00
Dyetrose	132.00	132.00	132.00
Cornstarch	397.49	397.49	397.49
Sucrose	100.00	100.00	100.00
Cellulose	50.00	50.00	50.00
t-Butylhydroquinone (TBHQ)	0.01	0.01	0.01
AIN 93-G Salt Mix	35.00	35.00	35.00
AIN-93G Vitamin Mix (with vitamin E)	10.00	10.00	10.00
LCystiene	3.00	3.00	3.00
Choline Bitartrate	2.50	2.50	2.50
Corn Oil^a^^,^ b	70.00	10.00	10.00
High-Oleic Safflower Oil^a^^,^ c	-	60.00	-
DHA-Enriched Fish Oil^a^^,^ d	-	-	60.00
**Unsaturated fatty acid composition**			
n-3 (DHA plus EPA)	0	0	35.4
n-6 (linoleic acid)	42.8	14.5	6.1
n-9 (oleic acid)	18.2	47.6	2.6

a As reported by the manufacturer.

b Corn oil contained 612 g/kg linoleic acid and 26 g/kg of oleic acid.

c High oleic acid safflower oil contained 140 g/kg linoleic acid and 750 g/kg oleic acid.

d DHA-enriched fish oil contained 540 g/kg DHA and 50 g/kg EPA.

### Experimental Design

Beginning at 4 wk of age, mice (n = 16 per group) were randomly assigned to DFO, CRN or HOS treatments and maintained on diet until sacrifice. Diets were prepared weekly and stored at −20°C to avoid oxidation. After assignment, all mice were further subdivided (n = 8 per group) into a pre-nephritis cohort that was terminated at 16 wk of age or a post-nephritis cohort that was terminated at 34 wk of age. Body weights were measured biweekly. Urine for proteinuria determination was obtained at regular intervals using the animal spot-urine collection method [31]. Blood was collected for plasma autoantibody and immunoglobulin analysis at selected intervals from the saphenous vein using lithium-heparin-treated microvettes (Sarstedt, Numbrecht, Germany) [32]. At termination, animals were euthanized via an injection of sodium pentobarbital (60 mg/kg body weight) and cervical dislocation. Terminal blood collection was made by cardiac puncture using heparinized (100 IU/ml) syringes. The left kidney was excised and immersed in 10% (v/v) neutral buffered formalin for histopathology. The right kidney and spleen were immersed in RNAlater (Ambion, Inc., Austin, TX) for mRNA analysis by PCR array and quantitative RT-PCR.

### Glomerulonephritis Assessment

Protein in urine was assessed utilizing Multistix’s 10 SG Urine Strips (Siemens Healthcare Diagnostics, Deerfield, IL). Readings greater than 300 mg/dL were considered indicative of proteinuria. For histopathological analysis, formalin-fixed paraffin-embedded kidneys were sectioned at 5 µm and stained with either Hematoxylin and Eosin [H&E] or Periodic acid Schiff [PAS]. Identifications were masked and glomerular injury was scored blindly by a board certified veterinary pathologist using the International Society of Nephrology-Renal Pathology Society Lupus Nephritis Classification [33].

### Measurement of Anti-dsDNA Autoantibodies and Immunoglobulins in Plasma

Whole heparinized blood was centrifuged at 2000×g for 5 min and the resultant plasma was collected and stored at −80C until analysis. Plasma anti-dsDNA IgG autoantibodies were determined using dsDNA ELISA kits (Alpha Diagnostic International, San Antonio, TX). Total IgM, IgG1, IgG2a, IgG2b, IgG3, IgA was assessed utilizing a Milliplex bead assay (Millipore Corp., Billerica, MA).

### Analysis of CD4^+^T Cell-related mRNA Expression in Kidney and Spleen by Focused PCR Array

Kidney and spleen samples were held in RNAlater at 4°C overnight and then stored at −80°C until analysis. Extraction of total renal and splenic RNA was performed with TriReagent (Sigma Aldrich Co., St. Louis, MO) according to manufacturer instructions. Genomic DNA was removed employing an RNeasy Mini Kit with DNase treatment (Qiagen Valencia, CA). Total RNA was dissolved in Ambion nuclease-free water and quantified using a NanoDrop-1000 (Thermo Fisher Scientific, Wilmington, DE). The SA Biosciences Mouse Profiler Th1-Th2-Th3 PCR Array (Frederick, MD) was used to assess impact of dietary treatments on CD4^+^ T cell-related mRNA expression. Prior to array analysis, samples were pooled (n = 8) using equal concentrations of RNA. cDNAs were prepared and arrays loaded according to manufacturer instructions. PCR was conducted using ABI 7900 HT Fast Real-Time PCR System (Foster City, CA) under universal RNA cycling conditions (10 min at 95°C, 15 s at 95°C, 1 min 60°C for 40 cycles). Data analysis was performed using SA Bioscience’s proprietary online program (http://pcrdataanalysis.sabiosciences.com/pcr/arrayanalysis.php, (last accessed 2/19/2014) which utilizes the ΔΔCt method for reporting expression changes.

### Quantitative PCR of Selected CD4^+^ T Cell-related mRNAs in Kidney and Spleen

Quantitative RT-PCRs using specific primer-probes obtained from Applied Biosystems (Foster City, CA) were performed on selected genes that were identified in the array study (CD80, CTLA-4, IL-10, IL-18, CCL-5, CXCR3, IL-6, TNF-α and OPN). Data were analyzed with ABI 7900 HT Fast Real-Time PCR System Applied Biosystems SDS 2.3 software and the absolute quantification method [34]. HPRT1 was used as the housekeeping gene. Data were normalized to expression of the target gene at wk 16 in the HOS-fed group.

### Statistics

Data are presented as mean +/− standard error of the mean (SEM). Statistical differences (p-value<0.05) were assessed using one-way ANOVA (Systat Software, Inc., San Jose, CA) and Tukey post hoc test for multiple comparisons. When normality or equality of variance failed, the Kruskal-Wallis ANOVA and/or Dunn’s post hoc test was applied.

## Results

### DFO Consumption Suppresses Glomerulonephritis and Body Weight Increase

**Figure 1:**
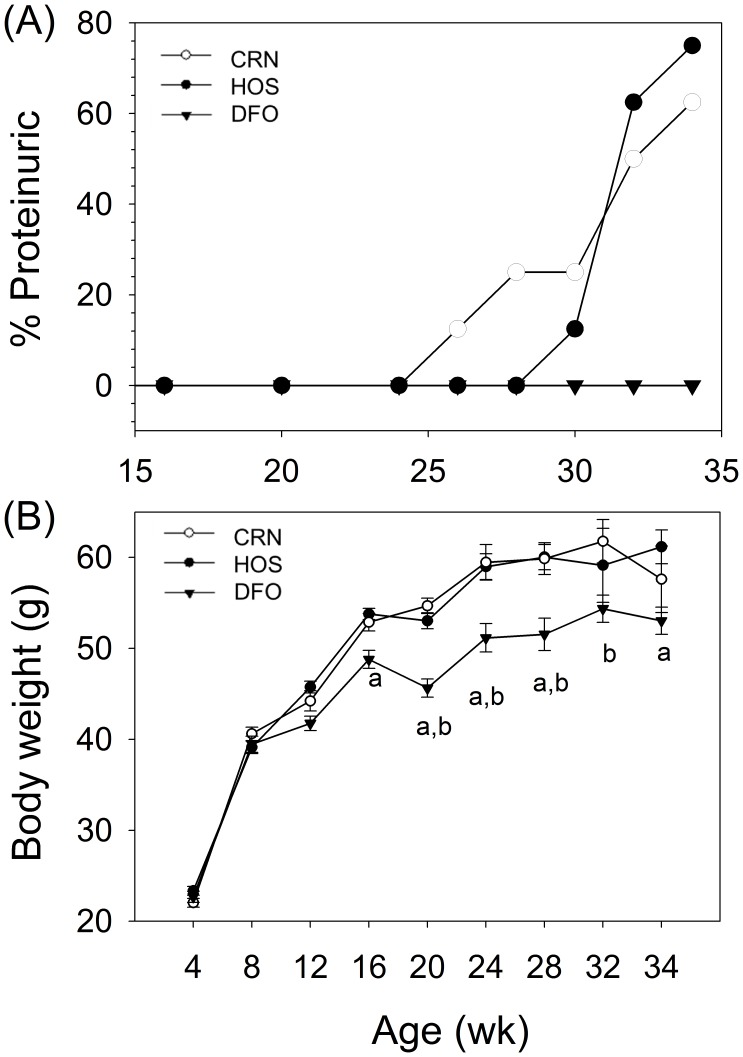
n-3 PUFA consumption prevents proteinuria onset and reduces weight gain. Female NZBWF1/J mice were fed DFO, CRN or HOS diets beginning at 4 wk of age. (A)Urinary protein was assessed using urinalysis reagent strips. Mice exhibiting ≥300 mg/dL were considered positive for proteinuria. (B) Effect of experimental diets on body weight. Values are mean ± SEM (n = 16) until 16 wk of age and n = 8, thereafter Letters *a* and *b* indicate body weights of DFO-fed mice differ significantly from those of CRN- and HOS-fed mice, respectively (p<0.05).

The effects of consuming DFO, CRN and HOS diets enriched in n-3, n-6 and n-9 unsaturated fats, respectively, on proteinuria, an indicator of glomerulonephritis, were compared in female NZBWF1 mice. Proteinuria was initially observed in 13% of the CRN-fed animals at wk 26 and this increased to 63% by wk 34 (Fig. 1A). The HOS treatment group exhibited proteinuria in 13% of the mice beginning at wk 30 which increased to 75% by wk 34. In contrast to these two groups, proteinuria was not observed in mice that consumed the DFO diet up to and including wk 34. Interestingly, HOS- and CRN-fed mice began to exhibit greater body weights than the DFO-fed group beginning at 20 wk of age (Fig. 1B). Upon termination at the age of 34 wk, DFO-fed mice weighed 8 and 12% less than HOS- and CRN-fed animals, respectively.

**Figure 2:**
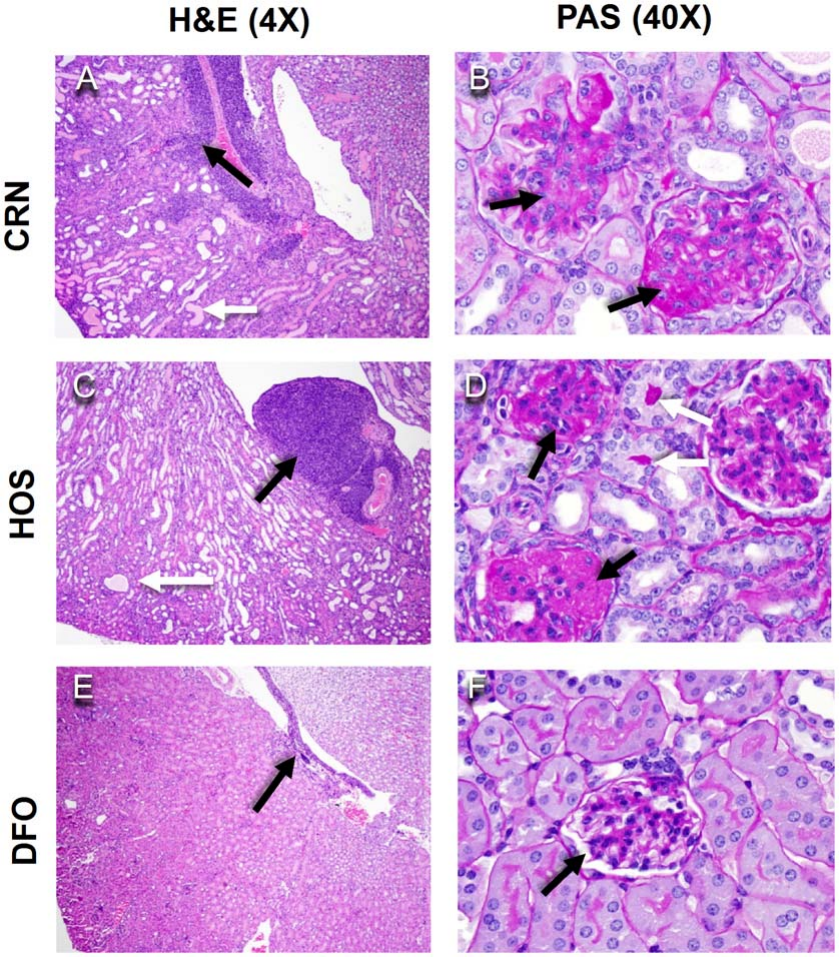
n-3 PUFA consumption attenuates glomerulonephritis. Female NZBWF1/J mice were fed CRN, HOS, or DFO diets for 30 wk beginning at 4 wk. At wk 34, renal histopathology was assessed. Panels A, C and F show representative hematoxylin and eosin (H&E)-stained sections (4X). Note the variable degree of tubular dilation and proteinosis (white arrow) and lymphocyte infiltration at the pelvis (black arrows). Panels B, D and F depict Periodic Acid-Schiff (PAS)-stained sections (40X). Note the tubular proteinosis (white arrows) and the thickened tubular basement membrane and mesangial cell hyperplasia-related glomerular hypercellularity with extensive adhesions to the Bowman’s capsule (black arrows). Lesions were moderate to severe in mice fed CRN and HOS diets and were mild in the mice fed DFO diet.

**Figure 3:**
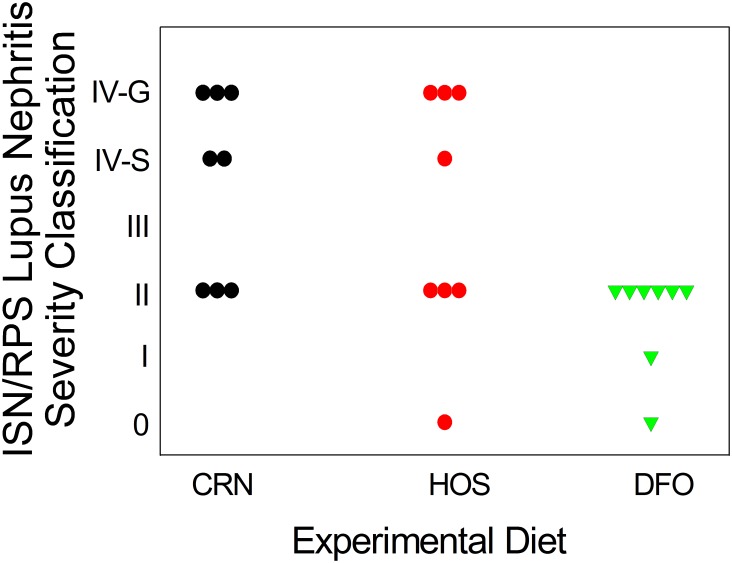
Summary of histopathological findings. Figure depicts summary of histopathological findings using ISN/RPS lupus nephritis classification system.

To confirm effects of the three dietary regimens on glomerulonephritis, kidney sections from the 34 wk cohort were assessed histopathologically following H&E and PAS staining. Both CRN and HOS fed mice exhibited: 1) moderate to severe diffuse glomerular hypercellularity, 2) mesangial expansion, and 3) tubular proteinosis characteristic of glomerulonephritis. Additionally, the mice had moderate to severe lymphocytic inflammation at the renal pelvis (Fig. 2A–D). In contrast, these endpoints were modest or absent in DFO-fed mice (Fig. 2E, F). Mice were individually graded for severity of lupus nephritis lesions using the ISN-RPS classification system. 5 out of 8 mice in the CRN group and 4 out of 8 in the HOS group had lesions categorized as class IV, which is indicative of severe glomerulonephritis (Fig. 3). In comparison, all DFO-fed mice had lesions classified as II or lower. Thus, consistent with proteinuria data, consumption of n-3 PUFA attenuated both the onset and severity of glomerulonephritis as compared to the n-6 PUFA or n-9 MUFA diet groups.

### DFO Dietary Regimen Inhibits Autoantibody Production

**Figure 4:**
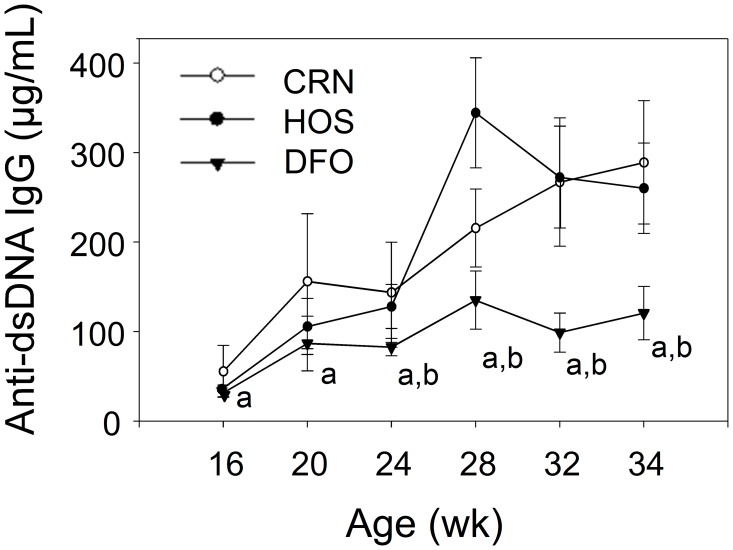
n-3 PUFA consumption delays autoantibody production. Female NZBWF1/J mice were fed CRN, HOS, or DFO diets beginning at 4 wk of age. Plasma anti-dsDNA autoantibody formation was monitored by ELISA. Data are expressed as mean ± SEM (n = 8). Letters *a* and *b* denote significant differences (p<0.05) from CRN- and HOS-fed mice, respectively.

Elevation of circulating autoantibodies is known to precede and/or co-occur with nephritis in NZBWF1 mice and is a key contributing factor in human SLE [3]. Consistent with this notion, mice fed CRN and HOS diets exhibited elevated anti-dsDNA IgGs in plasma beginning at wk 20 to 24, with concentrations increased 6- and 7-fold from wk 16 to wk 34, respectively (Fig. 4). By comparison, plasma anti-dsDNA IgG concentrations in the DFO treatment group were significantly lower (P<0.05) than those in the CRN and HOS treatment groups at all weeks tested.

**Figure 5:**
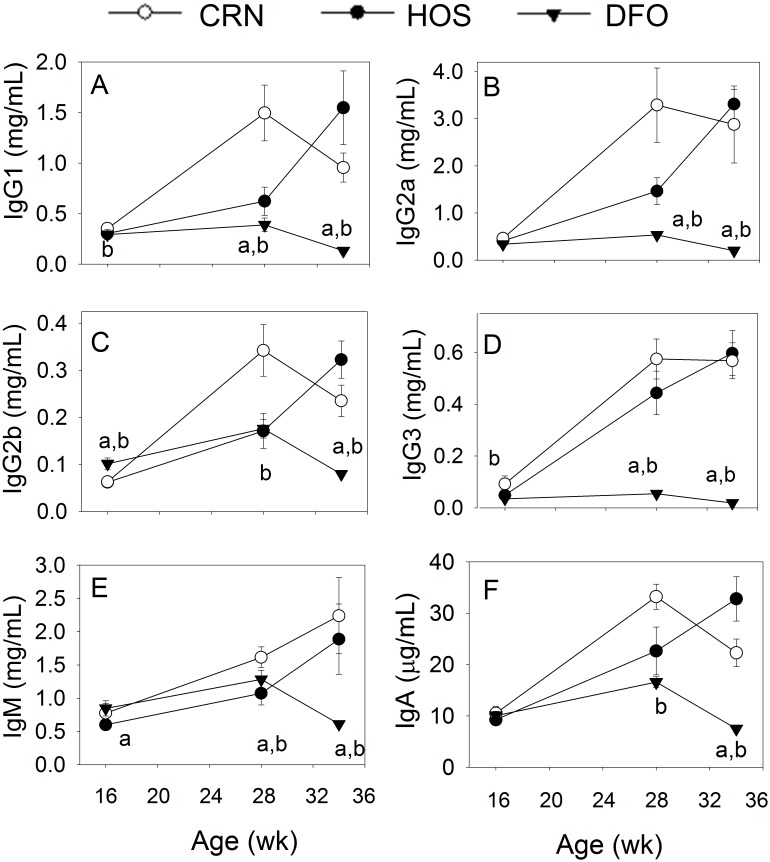
n-3 PUFA consumption inhibits plasma IgG1, IgG2a, IgG2b, IgG3, IgM and IgA elevation. Female NZBWF1/J mice were fed CRN, HOS, or DFO diets beginning at 4 wk of age**.** Concentrations of immunoglobulin isotypes were measured at wks 16 (n = 16), 28 (n = 8) and 34 (n = 8) using Milliplex Bead assay. Data are expressed as mean ± SEM. Letters *a* and *b* denote significant differences (p<0.05) from CRN- and HOS-fed mice, respectively.

Since polyclonal B cell activation and differentiation to IgG-secreting plasma cells occurs during SLE [35], [36], the effects of the different dietary regimens on plasma Ig isotype levels were also assessed. Compared to wk 16, concentrations of plasma IgG1, IgG2a, IgG2b, IgG3, IgM and IgA were increased by 2- to 12-fold at wk 34 in the CRN and HOS treatment groups (Fig. 5A–F). In contrast, concentrations of all isotypes in the DFO treatment group were significantly lower (p<0.05) than the CRN and HOS groups at wk 34.

### DFO Consumption Downregulates Expression of CD4^+^ T Cell-related Genes in Kidney and Spleen

**Figure 6:**
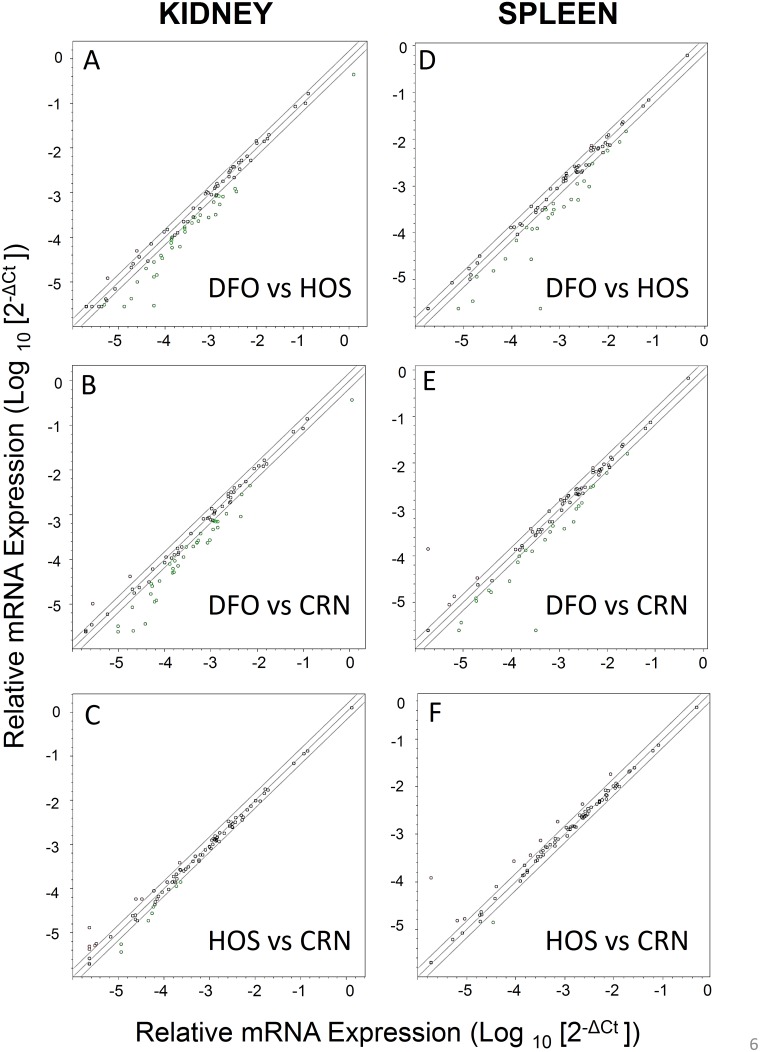
Comparative effects of n-3 PUFA consumption on CD4^+^ T cell-related gene expression in kidneys and spleens of 34 wk old NZBWF1/J mice. Cohorts of female mice were fed CRN, HOS, or DFO diets for 30 wk beginning at 4 wk. At wk 34, mice were euthanized and mRNA isolated from kidneys. Extracted mRNA were pooled and analyzed using a SABioscience Mouse Th1-Th2-Th3 PCR Array. Points outside of solid line are >1.5–fold difference.

Relative expression of 84 CD4^+^-T cell-related genes in kidneys and spleens from the three treatment groups were compared at wk 16 and wk 34 using a proprietary mouse profiling PCR array. While differences among groups at wk 16 were modest (see Fig. S1 and Table S1), they were markedly robust at wk 34 (Fig. 6, Table 2). A total of 33 genes on the array were found to be downregulated ≥1.5-fold while 3 were upregulated ≥1.5 fold in kidneys of DFO-fed mice as compared to HOS-fed mice (Fig. 6A). A similar comparison of DFO-fed mice with CRN-fed mice identified 37 downregulated and 2 upregulated genes (Fig. 6B). In spleens of 34 wk old mice, 26 genes were downregulated and 1 upregulated at 34 wk in DFO-fed mice as compared to HOS-fed mice. Similarly, 25 genes were downregulated and 7 were upregulated as compared to CRN-fed mice (Fig. 6D, E). In contrast to the aforementioned effects, comparison of CD4^+^ T cell-related gene expression between the HOS and CRN group revealed more modest differences with 7 and 8 genes being downregulated and upregulated, respectively, in kidney, (Fig. 6C) and 1 and 10 genes being downregulated and upregulated, respectively, in spleen (Fig. 6F).

**Table 2:** Differential effects of consuming n-3, n-6 and n-9 PUFAs on expression of CD4^+^ T cell-related genes in kidneys and spleens of 34 wk old female NZBWF1 mice^a^^,^ b.

Gene ID	Gene Description	KIDNEY			SPLEEN	
		DFO vs CRN		DFO vsHOS	DFO vsCRN	DFO vs HOS
**Integral Membrane Protein**						
Ccr2	Chemokine (C-C motif) receptor 2	−2.0		−2.2	1.3	1.4
Ccr3	Chemokine (C-C motif) receptor 3	−2.4		−2.8	1.5	1.6
Ccr4	Chemokine (C-C motif) receptor 4	−10.6		−4.4	−1.0	1.0
Ccr5	Chemokine (C-C motif) receptor 5	−2.1		−2.7	−2.2	−4.2
Ccr10	Chemokine (C-C motif) receptor 10	−1.3		1.3	−2.2	−2.2
Cd4	CD4 antigen	−2.3		−2.1	1.2	1.3
Cd27	CD27 antigen	−3.0		−2.0	1.2	−1.0
Cd28	CD28 antigen	−1.5		−1.7	−1.2	−1.1
Cd40	CD40 antigen	−1.6		−1.3	−1.2	−1.4
Cd40lg	CD40 ligand	−1.8		−2.6	−1.2	−1.0
Cd80	CD80 antigen	−5.4		−4.7	−1.2	1.0
Cd86	CD86 antigen	−1.6		−1.5	−1.2	−1.0
Ctla4	Cytotoxic T-lymphocyte-associated protein 4	−5.3		−4.5	−3.3	−3.3
Cxcr3	Chemokine (C-X-C motif) receptor 3	−2.1		−2.3	−2.2	−2.5
Icos	Inducible T cell co-stimulator	−2.1		−1.6	−1.6	−1.6
Igsf6	Immunoglobulin superfamily, member 6	−1.8		−1.8	−1.0	−1.1
Il1r1	Interleukin 1 receptor, type I	−1.6		−1.5	1.2	1.2
Il2ra	Interleukin 2 receptor, alpha chain	3.7		2.1	−1.4	−2.7
Il4ra	Interleukin 4 receptor, alpha	−2.9		−2.4	−130.0	−168.8
Il12rb2	Interleukin 12 receptor, beta 2	ND		−1.5	74.9	1.2
Il13ra1	Interleukin 13 receptor, alpha 1	−1.0		1.1	1.2	1.1
Il18r1	Interleukin 18 receptor 1	−1.8		−1.5	1.5	1.4
Il27ra	Interleukin 27 receptor, alpha	−1.6		−1.4	−1.1	−1.0
Ptprc	Protein tyrosine phosphatase, receptor type C	−3.5		−3.9	1.1	−2.0
Tlr4	Toll-like receptor 4	−1.4		−1.5	−1.0	−1.1
Tlr6	Toll-like receptor 6	−1.1		−1.8	1.1	1.1
Tmed1	Transmembrane emp24 domain containing 1	1.2		1.3	−1.0	−1.0
Tnfrsf4	Tumor necrosis factor receptor superfamily, member 4	−1.4		−1.6	−2.1	−2.1
Tnfrsf8	Tumor necrosis factor receptor superfamily, member 8	−4.1		−1.3	−1.9	−1.9
**Kinases**						
Jak1	Janus kinase 1		−1.2	−1.1	1.2	1.2
Jak2	Janus kinase 2		1.1	1.2	1.4	1.5
Jak3	Janus kinase 3		−1.1	1.0	−1.5	−1.5
Junb	Jun-B oncogene		−2.1	−1.5	−1.2	−2.7
Mapk8	Mitogen-activated protein kinase 8		1.3	1.2	1.5	1.2
Mapk9	Mitogen-activated protein kinase 9		1.2	1.3	1.2	1.2
Tyk2	Tyrosine kinase 2		−1.0	1.0	−1.0	1.1
Cytokine/Chemokine						
Ccl5	Chemokine (C-C motif) ligand 5		−4.9	−3.4	−1.4	−1.5
Ccl7	Chemokine (C-C motif) ligand 7		−2.7	−1.9	−3.3	−9.6
Ccl11	Chemokine (C-C motif) ligand 11		2.4	2.0	−1.9	1.3
Csf2	Colony stimulating factor 2(granulocyte-macrophage)		ND	ND	ND	ND
Ifng	Interferon gamma		−8.3	−19.5	−1.5	−2.7
Il2	Interleukin 2		ND	−4.7	−1.5	−1.7
Il4	Interleukin 4		1.0	−1.2	−1.8	−1.4
Il5	Interleukin 5		−1.2	−1.4	1.2	1.2
Il6	Interleukin 6		−1.5	1.3	−2.3	−2.7
Il7	Interleukin 7		1.1	1.4	−1.1	−1.2
Il9	Interleukin 9		1.1	1.4	ND	ND
Il10	Interleukin 10		−3.1	−3.4	−3.7	−4.8
Il12b	Interleukin 12B		−2.1	−1.5	−1.1	−1.4
Il13	Interleukin 13		1.3	−1.6	1.8	1.5
Il15	Interleukin 15		1.0	1.2	1.2	1.2
IL17a	Interleukin 17A		ND	ND	ND	ND
Il18	Interleukin 18		−2.0	−1.9	1.0	1.1
Il23a	Interleukin 23, alpha subunit p19		1.1	1.3	−2.5	−4.6
Il27	Interleukin 27		−1.2	−2.1	−1.5	−1.4
Opn	Osteopontin		−3.1	−2.9	−2.0	−2.4
Tgfb3	Transforming growth factor, beta 3		−1.3	−1.5	−1.1	−1.6
Tnf	Tumor necrosis factor		−2.3	−2.1	−1.2	−1.4
Tnfsf4	Tumor necrosis factor superfamily, member 4		−3.1	−1.4	1.1	1.3
**Transcription Factors and Regulators**						
Bcl6	B cell leukemia/lymphoma 6		−1.1	1.1	−1.4	−1.4
Cebpb	CCAAT/enhancer binding protein (C/EBP), beta		−1.1	−1.0	−1.6	−1.7
Crebbp	CREB binding protein		1.1	1.1	1.2	1.3
Gata3	GATA binding protein 3		−1.3	−1.1	−1.1	−1.2
Gfi1	Growth factor independent 1		−2.4	−2.6	−1.2	−1.7
Irf1	Interferon regulatory factor 1		−1.6	−1.2	−1.4	−1.3
Irf4	Interferon regulatory factor 4		−2.7	−1.7	−1.1	1.0
Maf	V-maf AS42 oncogene homolog		1.1	1.5	−1.2	−1.2
Nfatc1	NF of activated T cells, cyto., calcineurin-dep. 1		−1.2	−1.0	−1.1	1.2
Nfatc2	NF of activated T cells, cyto., calcineurin-dep. 2		−1.0	1.1	−1.2	−1.0
Nfatc2ip	Natc2 interacting protein		1.0	1.1	1.3	1.3
Nfatc3	NF of activated T cells, cyto., calcineurin-dep. 3		1.2	1.3	1.1	1.2
Nfkb1	NF of Κ light PP gene enhancer in B cells 1, p105		−1.4	−1.1	1.0	1.3
Pcgf2	Polycomb group ring finger 2		1.3	−1.3	ND	ND
Stat1	Signal transducer and activator of transcription 1		−1.6	−1.4	−1.7	−1.6
Stat4	Signal transducer and activator of transcription 4		ND	1.1	2.1	−1.1
Tbx21	T-box 21		−1.2	1.1	−2.3	−2.8
Tcfcp2	Transcription factor CP2		−1.0	1.3	1.5	1.4
Yy1	YY1 transcription factor		1.3	1.3	1.1	1.2
**Miscellaneous**						
Inha	Inhibin alpha		−1.2	−1.1	1.7	1.5
Il18bp	Interleukin 18 binding protein		−1.1	1.1	−1.1	−1.2
Socs1	Suppressor of cytokine signaling 1		−1.6	−1.5	−1.7	−1.8
Socs3	Suppressor of cytokine signaling 3		−3.6	−3.2	−1.3	−3.4
Socs5	Suppressor of cytokine signaling 5		1.2	1.2	−1.0	1.1
Sftpd	Surfactant associated protein D		−2.4	−1.9	−3.3	−3.4

a For qRT-PCR array comparisons, RNA expression values obtained from the kidneys of female NZBWF1/J mice fed docosahexaenoic acid-enriched diets were made relative to specified feeding group values and expressed as fold change. Expression ≥1.5 fold change was considered noteworthy.

b Abbreviations are: CRN, corn oil-enriched diet; HOS, high-oleic safflower oil-enriched diet; DFO, docosahexaenoic acid ethyl ester-enriched diet; ND, not detected.

Overall, expression of many CD4^+^ T cell-related genes was suppressed by DFO consumption at wk 34 (Table 2). These included 1) cytokines (IFN-γ, IL-2, IL-10, IL-18, IL-27, TNF-α, osteopontin [OPN]), 2) chemokines (CCL5, CCL7), 3) chemokine receptors (CCR2, CCR3, CCR4, CCR5, CXCR3), 4) T cell membrane proteins (CD4, CD28, CD40L, CTLA 4) and 5) accessory and B cell membrane proteins (CD27 and CD80). As described in the following sections, representatives from each of these gene families were selected and their mRNAs measured by quantitative real-time PCR in kidney and spleens at 16 and 34 wks.

### DFO Treatment Downregulates CD80 and CTL4A mRNA Expression in Kidney and Spleen

**Figure 7:**
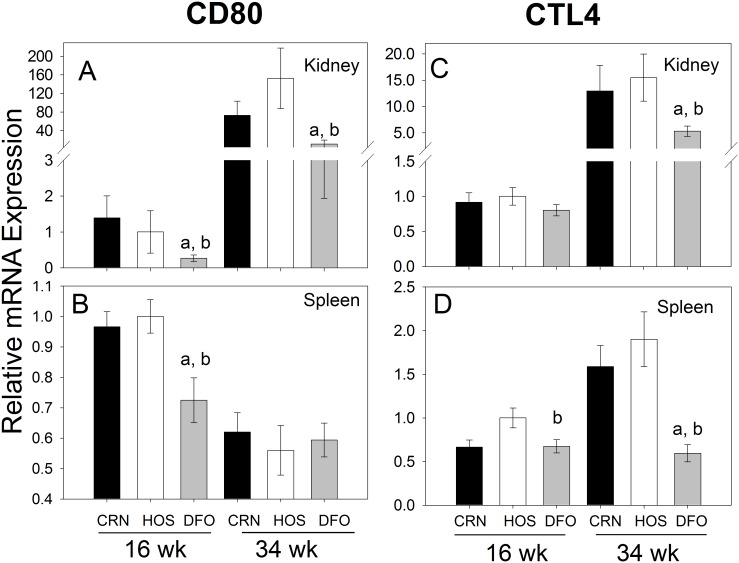
Differential effects of n-3, n-6 and n-9 PUFA consumption on CD80 and CTLA4 mRNA expression. Cohorts of 4/J mice were fed diets enriched in n-3 (DFO), n-6 (CRN) or n-9 (HOS) for 30 wk. At wk 16 (pre-nephritis) and wk 34 (post-nephritis), mRNA was isolated from (A, C) kidneys and (B, D) spleens and analyzed for gene expression of CD80 (A, B) and CTLA4 (C, D) by real-time PCR. Data are expressed as mean ±SEM (n = 8). Letters *a* and *b* denote significant differences (p<0.05) from CRN- and HOS-fed mice, respectively.

CD80 and CTL4A are integral membrane proteins on antigen-presenting cells and CD4^+^ T cells, respectively, that were downregulated in the array of DFO fed mice compared to either CRN- or HOS-fed mice. Quantitative RT-PCR indicated that, in the kidney, expression of CD80 mRNA in the CRN and HOS treatment groups increased by 52- and 153-fold, respectively from wk 16 to wk 34 (Fig. 7A). However, CD80 mRNA expression in the DFO treatment group was attenuated at both wk 16 (2- to 3-fold) and wk 34 (5- to 15-fold) as compared to CRN and HOS groups. CD80 expression was also modestly suppressed in the spleens of DFO-fed mice at wk 16 but not wk 34 (Fig. 7B). CTLA-4 mRNA expression in kidney increased from wk 16 to wk 34 by 14- and 15-fold in CRN- and HOS-treated mice, respectively (Fig. 7C), while DFO treatment attenuated these responses by 2- to 3-fold. Although increases in splenic CTLA4 mRNA expression from wk 16 to wk 34 were more modest than kidney, relative mRNA levels for this gene at wk 34 were again 2- to 3-fold lower in the DFO group than the CRN and HOS groups (Fig. 7D).

### Expression of IL-10 and IL-18 mRNA in Kidney and Spleen is Attenuated following N-3 PUFA Consumption

**Figure 8:**
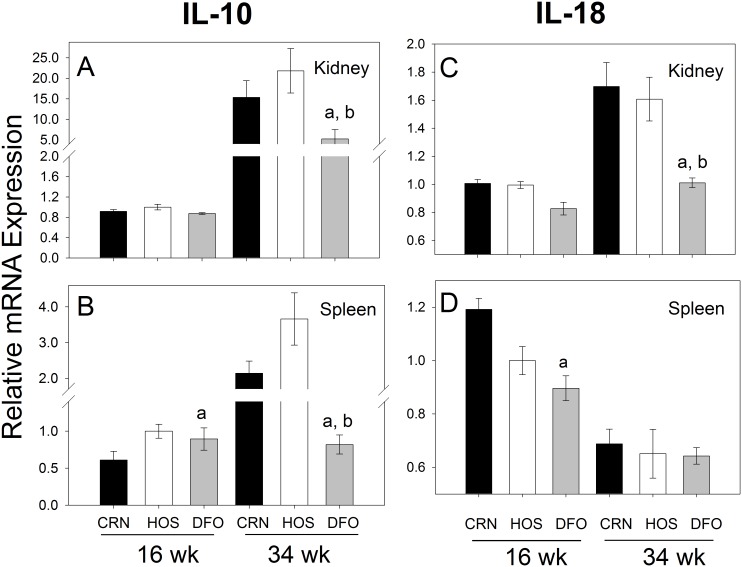
Differential effects of n-3, n-6 and n-9 PUFA consumption on IL-10 and IL-18 mRNA expression. Experiment conducted as described in Fig. 7 legend except that IL-10 (A, B) and IL-18 (C, D) mRNAs were measured by quantitative RT-PCR.

IL-10 and IL-18 are examples of regulatory cytokines that impact T cell function, both of which were downregulated in the PCR array. Quantitative RT-PCR revealed that IL-10 mRNA expression in kidney robustly increased from wk 16 to wk 34 in CRN and HOS treatment groups by 17- and 22-fold, respectively (Fig. 8A), whereas IL-18 mRNAs for these groups increased more modestly (Fig. 8C). Elevated expression at wk 34 of both genes in the kidney was significantly attenuated in the DFO treatment group. Consumption of CRN and HOS elevated IL-10 mRNA expression from 16 to 34 wk by 2- to 3-fold (Fig. 8B) in spleen. Consistent with the kidney, increases in IL-10 mRNA were largely ablated in mice that consumed DFO. However, dietary unsaturated fat regimens largely did not affect IL-18 mRNA expression in spleen (Fig. 8D).

### DFO Diet Suppresses CCL5 and CXCR3 mRNA Expression in Kidney and Spleen

**Figure 9:**
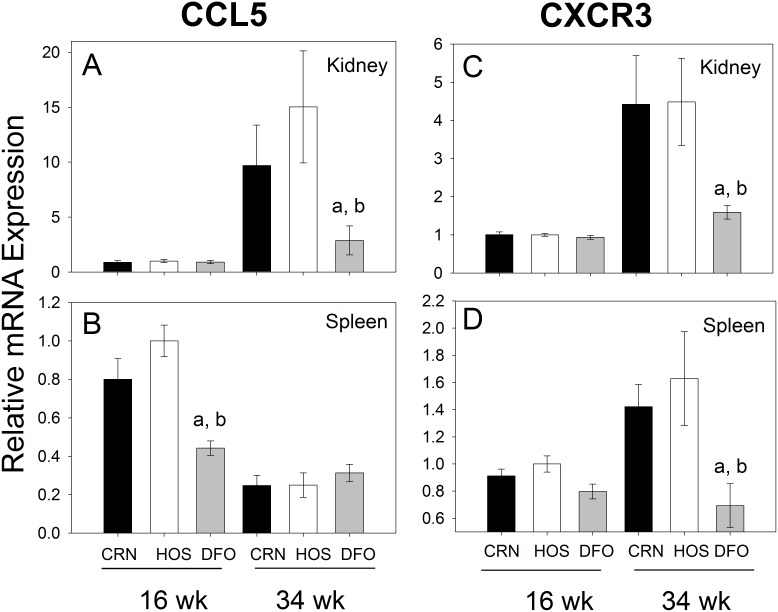
Differential effects of n-3, n-6 and n-9 PUFA consumption on CCL5 and CXCR3 mRNA expression. Experiment conducted as described in Fig. 7 legend except that CCL5 (A, B) and CXCR3 (C, D) mRNAs were measured by quantitative RT-PCR.

Downregulated expression of CCL5 and CXCR3 mRNAs, prototypical examples of a chemokine and chemokine receptor, respectively, in DFO-treated mice was verified by quantitative PCR. CCL5 mRNA was found to increase from wk 16 to 34 by 11- and 15-fold in the kidneys of CRN and HOS fed mice, respectively and these responses were suppressed in DFO fed mice by 4- to 7-fold (Fig. 9A). Splenic expression of this chemokine at 16 wk was also lower in DFO treatment animals than in CRN or HOS treatment groups, whereas no effects were observed at 34 wk (Fig. 9B). CXCR3 mRNA expression in the kidney also increased from 16 to 34 wk in mice fed CRN (4-fold) and HOS (6-fold) diets, but this elevation was suppressed by 3-fold in those mice fed DFO (Fig. 9C). Similar but more modest effects were observed in the spleen (Fig. 9D).

### DFO Consumption Impairs IL-6 and TNF-α mRNA Expression in Kidney and Spleen

**Figure 10:**
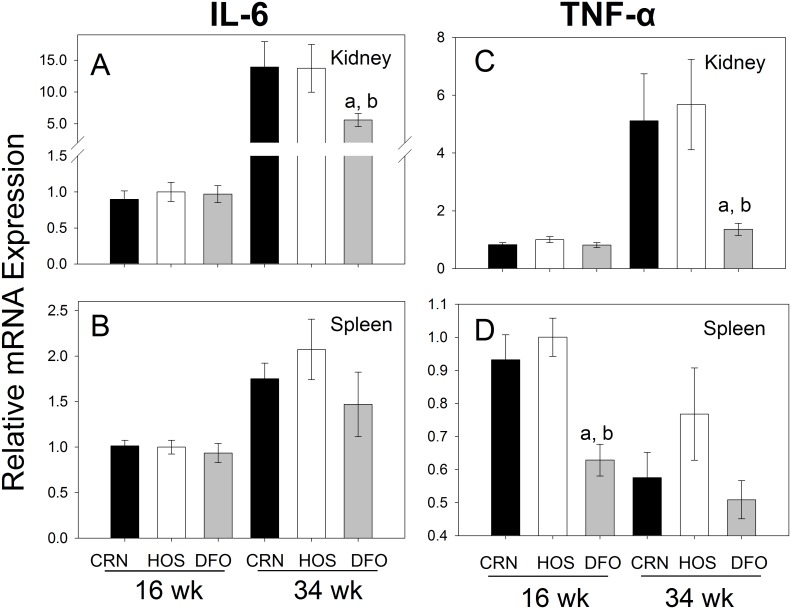
Differential effects of n-3, n-6 and n-9 PUFA consumption on IL-6 and TNF -α **mRNA expression.** Experiment conducted as described in Fig. 7 legend except that IL-6 (A, B) and TNF-α (C, D) mRNAs were measured by quantitative RT-PCR.

The proinflammatory cytokines IL-6 and TNF-α were markedly upregulated from wk 16 to 34 in the kidneys of mice consuming the CRN (6- and 15-fold, respectively) and HOS (6- and 14-fold, respectively) diets (Fig. 10A, C). In contrast, consumption of the DFO diet attenuated expression of IL-6 and TNF-α mRNAs in the kidneys by 3- to 5-fold. In spleen, while dietary treatment effects on IL-6 mRNA expression were not evident (Fig. 10B), DFO consumption modestly suppressed TNF-α mRNA expression at 16 wk but not at 34 wk (Fig. 10D).

### Osteopontin mRNA Expression is Inhibited in Kidneys and Spleens of DFO-fed Mice

**Figure 11:**
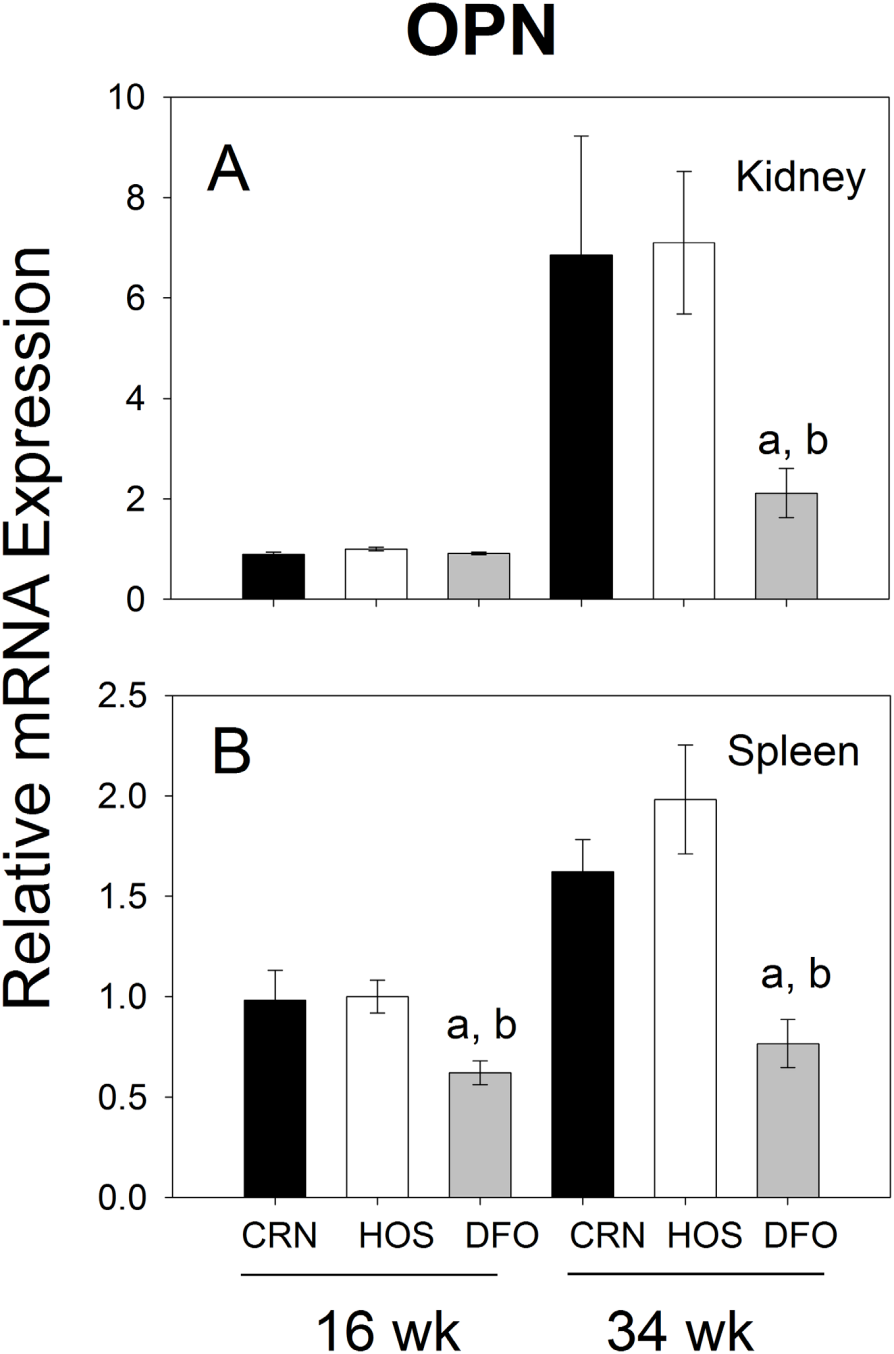
Differential effects of n-3, n-6 and n-9 PUFA consumption on OPN mRNA expression. Experiment conducted as described in Fig. 7 legend except that OPN mRNAs were measured by quantitative RT-PCR.

The dietary effects of n-3 PUFAs on expression of the pleotropic cytokine OPN were measured by quantitative PCR. In the kidney, mRNA levels of this gene increased from wk 16 to wk 34 by 7- to 8-fold in CRN- and HOS-fed mice, whereas these elevations were attenuated by DFO-feeding (Fig. 11A). In analogous fashion, DFO treatment reduced expression of osteopontin mRNA in the spleen at both wk 16 and wk 34 when compared to CRN or HOS treatment groups (Fig. 11B).

## Discussion

Preclinical models such as the female NZBWF1 mouse have been invaluable to unraveling the complex mechanisms involved in human SLE as well as identifying biomarkers and cellular targets for directing potential therapies. This chronic disease involves defects in the negative selection of autoreactive B and T cells as well as immune hyperactivity. Together these defects evoke aberrant activation of self-reactive lymphocytes, production of autoreactive antibodies and immune complex deposition in the kidney that result in glomerulonephritis. The CRN diet employed in this study was chosen to mimic the fatty acid profile of a typical Western diet in which individuals eat a high concentration of the n-6 PUFA linoleic acid, favoring formation of arachidonic acid and its inflammatory eicosanoids. The HOS diet was used to recapitulate consumption of high levels of oleic acid, primarily from olive oil, that are present in a Mediterranean diet. Consumption of this n-9 MUFA per se has been suggested to reduce inflammatory responses [37]. This diet also provided insight to whether simply reducing n-6 PUFA-derived arachidonic acid pools could be responsible for any observed protective effects of the n-3 PUFA diet. The results presented herein demonstrate that autoantibody production, polyclonal B cell activation and lupus nephritis were similarly evident in mice fed diets rich in n-6 or n-9 unsaturated fats. However, the onset and/or severity of these responses were markedly suppressed in mice consuming the n-3 PUFA diet. Thus, the effects of n-3 PUFAs could not be replicated by substitution with the n-9 MUFA oleic acid.

The focused PCR array study revealed that n-3 PUFA suppression of the autoimmune responses co-occurred with generalized downregulation of gene expression in kidney and/or spleen of CD4^+^ T cell-related genes associated with inflammation including leukocyte recruitment, antigen presentation, T cell activation, and B cell activation/differentiation. Furthermore, effects of replacing n-6 PUFAs with the n-9 MUFAs on gene expression responses were negligible. Downregulation of these genes by n-3 PUFAs in kidney and spleen could be explained by inherently lower expression by cells that populate these tissues and/or by decreased infiltration by T cells, B cells, antigen-presenting cells and other leukocytes. Importantly, genes downregulated by the n-3 PUFA regimen included CD80, CTLA-4, IL-10, IL-18, CCL-5, CXCR3, OPN, IL-6 and TNF-α. Many of these are under consideration as biomarkers and/or potential targets for specific monoclonal antibody or receptor antagonist treatments of SLE and other autoimmune diseases.

n-3 PUFAs likely exert the pleiotropic effects observed here via both eicosanoid-dependent and eicosanoid-independent pathways [10]. Eicosanoids are a diverse group of chemical mediators produced by immune cells that depend upon the cell membrane PUFA composition [38]. For example, the eicosanoid prostaglandin E2 (PGE2), a cyclooxygenase (COX) metabolite of AA, can be proinflammatory and modulate cytokine production. Likewise, the 4-series leukotrienes (LTs) are lipooxygenase (LOX) metabolites of AA that have chemotactic properties, and upregulate proinflammatory cytokine production promoting inflammation. DHA and EPA competitively displace AA as a substrate for oxygenation by both COX-2 and 5-LOX. This reduces inflammation through eicosanoid-dependent pathways by: 1) decreasing membrane AA levels, 2) suppressing generation of proinflammatory eicosanoids (2 series PGs and 4-series LTs), 3) inhibiting COX-2 and 5-LOX expression and 4) promoting production of novel metabolites. Regarding the latter, studies over the last decade from the Serhan lab have shown that n-3 PUFAs convert to a novel series of lipid mediators termed specialized proresolving lipid mediators. These include resolvins, protectins, and maresins that can elicit, sometimes at very low concentrations, protective and beneficial effects in the resolution of inflammation [39]. Eicosanoid-independent mechanisms also play critical roles in n-3 PUFA suppression of gene expression. These include: 1) changes in membrane lipid/lipid raft composition that alter G-protein receptor or tyrosine-kinase linked receptor signaling [40], [41], 2) interference with membrane receptors such as the TLR family [42], 3) alteration of transcription factor activity or abundance as has been described for PPAR, LXR, NNF-4, NF-κβ, AP-1 and CREB [43], [44], [45]and 4) interference with activity of critical second messenger-regulated kinases such as PKA, PKC, CaMKII, AKT and mitogen-activated protein kinases [45], [46], [47].

Another important consideration is the potential for n-3 PUFA consumption to inhibit inflammagenic mediator production associated with the increased fat mass and metabolic changes in NZBWF1 mice. White adipose tissue is composed of not only adipocytes, but also cells of the innate and adaptive immune systems that collectively secrete numerous cytokines and chemokines [48] that could contribute to development of SLE. Indeed numerous characteristics of obesity and metabolic syndrome have been observed in 36 wk old NZBWF1 mice that included increased body weight, hypertension, elevated plasma leptin, insulin resistance, and central adiposity with macrophage infiltration [49]. A study in B6.Sle.1Sle2.Sle3 SLE-prone mice reported significantly worsened glucose tolerance, increased adipose tissue insulin resistance, increased β-cell insulin secretion, and increased adipocyte size compared with their respective B6 controls [50]. B cells isolated from the white adipose tissue of B6.SLE mice were skewed toward IgG production, and the level of IgG1 was elevated in the serum of SLE-prone mice. In the study presented herein, DFO-fed NZBWF1 mice weighed 8 and 12% less than HOS- and CRN-fed animals, respectively. Thus, reduced body weight and fat mass could be factors in reduced SLE severity observed in mice that consumed DFO. Although we did not specifically measure food intake or adiposity in this study, Bargut et. al [51] recently reported that B6 mice fed high fat fish oil diet exhibit reductions in body weight, adiposity index and plasma IL-6 as compared to mice fed high fat soybean oil diets. Importantly, no differences in food intake were observed between the two groups, and, furthermore, fish oil-fed mice exhibited higher energy expenditure. Thus, it might be speculated that reduced body weights in DFO-fed NZBWF1 mice in the present study resulted from lower feed efficiency than that for CRN- and HOS-fed animals and that this reduction contributed to reduced adipose tissue inflammation. It will therefore be critical in future studies to relate n-3 PUFA attenuation of autoimmunity to the potential effects on food intake, feed efficiency, metabolism, obesity and leukocyte infiltration/gene expression to adipose tissue.

CD80, also known as B7-1, is constitutively expressed on dendritic cells and is inducible in other antigen-presenting cells such as B cells and monocytes [52]. This integral membrane protein provides the requisite costimulatory signal for T cell activation and survival. CD80 works in tandem with CD86 to prime T cells by serving as ligand for CD28 and CTLA-4 on the T cell surface [53]. CD28 is upregulated early during T cell activation and its stimulation can: 1) augment and prolong IL-2 secretion by T cells as well as delay the onset of peripheral immune tolerance, 2) enable T cells to signal B cells to proliferate and differentiate into antibody-producing plasma cells, and 3) enhance other cognate costimulatory interactions between antigen-presenting cells and T cells thereby intensifying proinflammatory gene expression. While structurally similar to CD28, CTLA-4 differs because it is expressed after prolonged T cell activation and transduces an inhibitory signal to T cells rather than a stimulatory signal [54].

Several lines of evidence indicate that impairment of the CD80 costimulatory pathway attenuates autoimmune effects associated with SLE. Blockade of CD80 by monoclonal antibody has been shown in the pristane-induced lupus mouse model to attenuate both the inflammatory response and the severity of SLE hallmarks [55]. It has been further shown in a murine SLE model that following treatment with CTLA4-Ig (Abatacept), a recombinant fusion protein that contains the Fc fragment of human IgG1 and binds either to CD80 or CD86 with a much higher avidity than CD28, that there is a reduction in both numbers of autoreactive B cells and autoantibodies [56]. CTLA4-Ig has been shown in clinical studies to dampen the interaction between T and B lymphocytes, resulting in the attenuation of autoimmune-driven inflammation [57]. Accordingly, CD80 mRNA downregulation observed here in spleens at wk 16 and kidneys at wk 16 and wk 34 of DFO-fed mice compared to CRN- and HOS-fed mice is of profound significance, because it implies that DHA inhibits the capacity of antigen-presenting cells to stimulate T cells and evoke both humoral and cell-mediated autoimmune sequelae. These findings are consistent with decreased CD80 and CD86 expression in peripheral blood mononuclear cells found previously in fish oil-fed NZBWF1 mice [58]. While the observation that DFO treatment also suppressed upregulation of CTLA-4 mRNA expression in spleen and kidney appears to be counterintuitive to this notion, these findings might rather reflect generalized downregulation of T cell activation, with tolerance-inducing late-stage CTLA-4 upregulation being particularly vulnerable to the immunosuppressive effects of DHA.

IL-10 is produced by many types of leukocytes including regulatory T cells, macrophages, dendritic cells and B cells [59]. This protein suppresses the capacity of macrophages and dendritic cells to present antigen and stimulate T cells, thereby limiting and controlling subsequent T cell responses [60]. In B cells, IL-10 acts as a potent growth factor that induces class switching and differentiation into Ig-secreting plasma cells [61]. SCID mice injected with PBMCs from human lupus patients produced less IgG when treated with anti-IL-10 antibodies [62] and similarly-treated mice showed less renal impairment after IL-10 blockade [63]. The finding that IL-10 is present in higher concentrations in lupus patients than controls, and is elevated in SLE patients with active disease compared to inactive, further supports the notion that IL-10 contributes to lupus pathogenesis [64]. One clinical trial of IL-10 blocking antibodies as a therapy for lupus has been reported that suggests there were benefits from this approach [65]. Accordingly, reduced IL-10 expression in kidneys and spleens of mice fed DFO could be another contributing factor to the reduced autoantibody production and nephritis observed in that treatment group.

IL-18, a proinflammatory cytokine produced by dendritic cells and macrophages, exerts pleiotropic effects on T cells, dendritic cells and natural killer cells [66] [67]. IL-18 contributes to the onset and severity of lupus indicating a critical pathogenic role of this cytokine [68], [69]. Decreased IL-18 gene expression observed here at wk 34 in kidneys of DFO-fed mice as compared to HOS- or CRN-fed mice therefore might be a factor in reduced lupus manifestations. Relatedly, the Fernandes group found that NZBWF1 mice fed DHA-enriched fish oil exhibited reduced serum and kidney IL-18 responses following LPS-challenge as compared to corn oil-fed mice [70]. It is important to note that IL-18 is a potent inducer of IFN-γ which can both skew the Th response towards a Th1 pattern and promote inflammation in lupus [71]. Interestingly, we observed in the PCR array 8- to 19-fold and 2- to 3-fold decreases in IFN-γ mRNA expression in kidneys and spleens of the DFO treatment group, respectively (Table 2). In future studies, it will therefore be important to link the suppressive effects of n-3 PUFA on IL-18 to Th1 polarization and IFN-γ expression.

Chemokines produced in inflamed tissues, and corresponding chemokine receptors expressed on the surface of the leukocytes, are essential for leukocyte infiltration at sites of inflammation. A number of chemokine genes (CCL5, CCL7, CCL11) were downregulated in DFO-fed mice. CCL5, also known as RANTES (regulated on activation, normal T cell expressed and secreted), is a C–C chemokine that plays a role in regulating Th-cell cytokine production and leukocyte trafficking. CCL5 is expressed by renal epithelial and mesangial cells, activated T cells, airway epithelial cells, fibroblasts, platelets and lymphocytes [72]. Importantly, CCL5 induces both leukocyte migration and activation and mediates the recruitment of lymphoid cells such as CD4^+^ and CD8+ T cells and monocytes to sites of inflammation [73]. *In vitro* studies have confirmed CCL5 produced by cytokine-activated proximal tubular epithelial cells in kidney promote selective recruitment of activated T cells via receptors on these cells specific for CCL5 [74]. Plasma CCL5 concentrations are significantly elevated in SLE patients when compared with normal controls and correlate with autoantibody levels, indicating that this chemokine might be involved in SLE pathogenesis [75]. Thus, decreased expression of CCL5 is likely to contribute to attenuated autoimmune and nephritic responses observed in n-3 PUFA-fed mice.

Expression of several chemokine receptors (CXCR3, CCR2, CCR3, CCR4, CCR5, CCR10) was depressed in the kidneys and/or spleens of the DFO treatment group. CXCR3 is a G protein–coupled receptor superfamily member that is expressed on activated T cells [76]. Natural ligands to CXCR3 are CXCL9, CXCL10, and CXCL11. Both the receptor and its ligands are considered potential therapeutic targets for treatment of lupus nephritis. CXCR3 deficiency ameliorates T cell infiltration and nephritic damage in the MRL/lpr model of SLE [77]. Similarly, lack of CXCR3 also reduces renal T cell infiltrates and nephritis after induction of nephrotoxic serum nephritis in non-autoimmune C57BL/6 mice [78], [79]. It is further notable that plasma cells expressing CXCR3 also localize into inflamed kidneys of lupus mice [80].

In human patients with lupus nephritis, CXCR3 is similarly expressed on subpopulations of activated T cells and plasma cells [81], [82] as well as in a large proportion of CD4**^+^** T cells that infiltrate the kidney [83]. Both systemic and kidney levels of CXCR3’s cognate ligands are elevated in SLE patients, all of which correlate with disease activity [84], [85]. CXCR3^+^CD4^+^ T cells are significantly elevated in the urine of lupus patients with active nephritis flares and are a useful biomarker for renal disease activity [83]. CXCR3 is also expressed on subpopulations of memory B cells and plasma cells from lupus patients and it has been demonstrated that plasma cell precursors migrate toward gradients of CXCR3 ligands [86]. Taken together, reduced expression of CXCR3 in kidneys and spleens of DFO-fed NZBWF1 mice observed here might reflect decreased populations of activated T and/or B cells that mediate autoimmune sequelae and nephritis observed in this model.

IL-6, one of the initial cytokines investigated in SLE pathogenesis, is produced by the monocytes, fibroblasts and endothelial cells as well as by T and B cells [67]. n-3 PUFA consumption has recently been shown in NZBWF1 mice to decrease both kidney IL-6 mRNA expression and ex vivo IL-6 secretion by LPS-induced splenocytes [87]. IL-6 induces 1) CD4 + T cell differentiation, 2) B cell maturation into plasma cells, 3) antibody secretion, 4) production of acute phase proteins and 5) promotion of macrophage and osteoclast differentiation [88]. Studies in lupus-prone mice support an important role for IL-6 in evoking autoimmune sequelae [89]. IL-6 is found at increased levels in SLE patients [90], and furthermore, IL-6 and its receptors can serve as biomarkers to monitor disease activity and treatment response [91], [92]. IL-6 blockade significantly abrogates spontaneous immunoglobulin secretion by B cells isolated from SLE patients, which is restorable with exogenous IL-6 [90]. An anti-IL-6 receptor antibody, Tocilizumab, approved for use in rheumatoid arthritis, shows promise in treating lupus, with effects that seemed directed at autoantibody production [93]. Therefore, decreased IL-6 expression in kidneys of DFO-fed mice might further contribute to the decreased autoimmune and nephritic responses observed here.

TNF-α is expressed after the activation of macrophages and dendritic cells and can promote expression of many genes related to inflammation. TNF-α concentrations are elevated in both sera and renal tissue of lupus-prone mice and SLE patients alike and its levels correlate with disease activity and glomerulonephritis [94], [95]. Accordingly, decreased TNF-α expression in kidney and spleens of mice fed DFO might also be important in the downregulation of autoimmune sequelae.

This is the first report of decreased OPN expression in a model of SLE following n-3 PUFA supplementation. OPN, also known as secreted phosphoprotein 1 (SPP1), is a pluripotent cytokine that is secreted by dendritic cells, macrophages, T cells, and B cells [96], [97]. OPN can be induced by several inflammatory cytokines including IL-1β, TNF-α and IFN-γ [98]. Because OPN enhances macrophage migration, survival and cytokine production, it plays an important role in chronic inflammation [99], [100] and pathogenesis of autoimmunity [101]. OPN contributes to activation of dendritic cells, enhancement of Th1 and inhibition of Th2 cytokine expression [102]. Importantly, OPN is also a polyclonal B cell activator [103], [104]. In murine lupus nephritis, a specialized subset of macrophages known as alternatively activated macrophages, which participate in tissue repair, express OPN and mediate aggressive proliferative lesions with enhanced crescent formations [105]. OPN deficiency in autoimmune models causes delayed onset of polyclonal B cell activation [100], [106]. Anti-dsDNA antibodies arise spontaneously in mice that overexpress OPN and have no other genetic abnormalities contributing to autoimmune disease phenotype [103]. In further support of the role of OPN in lupus nephritis, humans with SLE and autoimmune-prone mice have increased OPN systemically and in tissue lesions that correlate with disease activity [107], [108]. Elevation of OPN in human SLE precedes increased cumulative disease activity and organ damage [101]. Accordingly, OPN is an important factor in lupus, making its downregulation in DFO-fed mice a highly relevant finding.

The n-3 PUFA (DHA+EPA) concentration used here, 35.4 g/kg, was selected because this approximate level has been previously demonstrated to be efficacious by our laboratory for ameliorating both deoxynivalenol-induced IgA nephropathy and ex vivo IL-6 expression [43], [109], [110]. We have shown that consumption of this dietary n-3 PUFA level by mice for 2 wk was sufficient to increase splenic n-3 PUFA content by 6-fold (from 2.7 to 16.3%) and reduce n-6 PUFA content by 2-fold (from 21.9 to 11.5%) compared to HOS-fed mice [111]. Further changes in the n-3 PUFA content of splenocytes were not observed after 4 or 6 wk of feeding. Relatedly, Halade and coworkers [112] used the same commercial source of DHA-enriched fish oil as here to prepare a diet containing an approximate n-3 PUFA concentration of 58.5 g/kg that effectively inhibited autoimmune nephritis in the NZBWF1 mouse.

Typical n-3 PUFA intake recommendations for healthy people range from 0.5 to 2 g/d, but higher levels of consumption ranging from 3 to 20 g/d have been used in clinical trials for SLE therapy [26], [27], [113]. The combined DHA and EPA concentration in the DFO diet employed in the present study would account for 8.2% of total energy intake. Upon extrapolation, a human consuming 2000 kcal/d (8.368 MJ/d) would require 18 g/d to correlate with the amount consumed in this experiment. Thus, while these concentrations exceed normal human diet recommendations in terms of energy percentage in the diet, they are consistent with high-end therapeutic use of (n-3) PUFAs. It will be nevertheless be desirable in future studies to determine the dose response effects of DFO on autoimmune nephritis and expression of the genes identified here.

## Conclusion

We have demonstrated in the NZBWF1 preclinical SLE mouse model that consumption of n-3 PUFA-enriched diet effectively blunted the marked autoantibody and nephritic responses that were observed in mice consuming either an n-6 PUFA-enriched Western-style or an n-9 MUFA-enriched Mediterranean-style diets. Remarkably, these attenuating effects co-occurred with generalized downregulation of CD4^+^ T cell-associated genes in the kidney and, to a lesser extent, in spleen that are associated with antigen presentation, T cell activation, leukocyte recruitment, B cell activation/differentiation and inflammatory responses. Many of these genes are under consideration as potential targets for development of expensive biological therapeutics for human SLE including monoclonal antibodies and receptor antagonists. Supplementation with n-3 PUFAs might be a lower cost alternative that could impact many of these therapeutic targets simultaneously. A major concern with this strategy would be whether doses needed to suppress autoimmunity and inflammation will adversely impact health by interfering with innate and adaptive immune responses to pathogens or neoplastic events [114]. Accordingly, further clarification is needed on the underlying mechanisms and dose-response effects of n-3 PUFAs on expression of genes associated with prevention and intervention of SLE in the NZBWF1 mouse and other autoimmune models.

## Supporting Information

Figure S1 **Comparative effects of n-3 PUFA consumption on CD4^+^ T cell-related gene expression in kidneys and spleens of 16 wk old NZBWF1/J mice.** Cohorts of mice were fed CRN, HOS, or DFO diets for 30 wk beginning at 4 wk. At wk 34, mice were euthanized and mRNA isolated from kidneys. Extracted mRNA were pooled and analyzed using a SABioscience Mouse Th1-Th2-Th3 PCR Array. Points outside of solid line are>1.5–fold difference.
(TIF)

Table S1 **Differential effects of consuming n-3, n-6 and n-9 PUFAs on expression of CD4+ T cell-related genes in kidneys and spleens of 16** **wk old female NZBWF1 mice.**
(DOCX)
