# Supplementary figures and images for: Comparative Effects of n-3, n-6 and n-9 Unsaturated Fatty Acid-Rich Diet Consumption on Lupus Nephritis, Autoantibody Production and CD4+ T Cell-Related Gene Responses in the Autoimmune NZBWF1 Mouse
Source: PLoS One. 2014 Jun 19;9(6):e100255. doi: 10.1371/journal.pone.0100255 (PMC4063768; doi:10.1371/journal.pone.0100255)

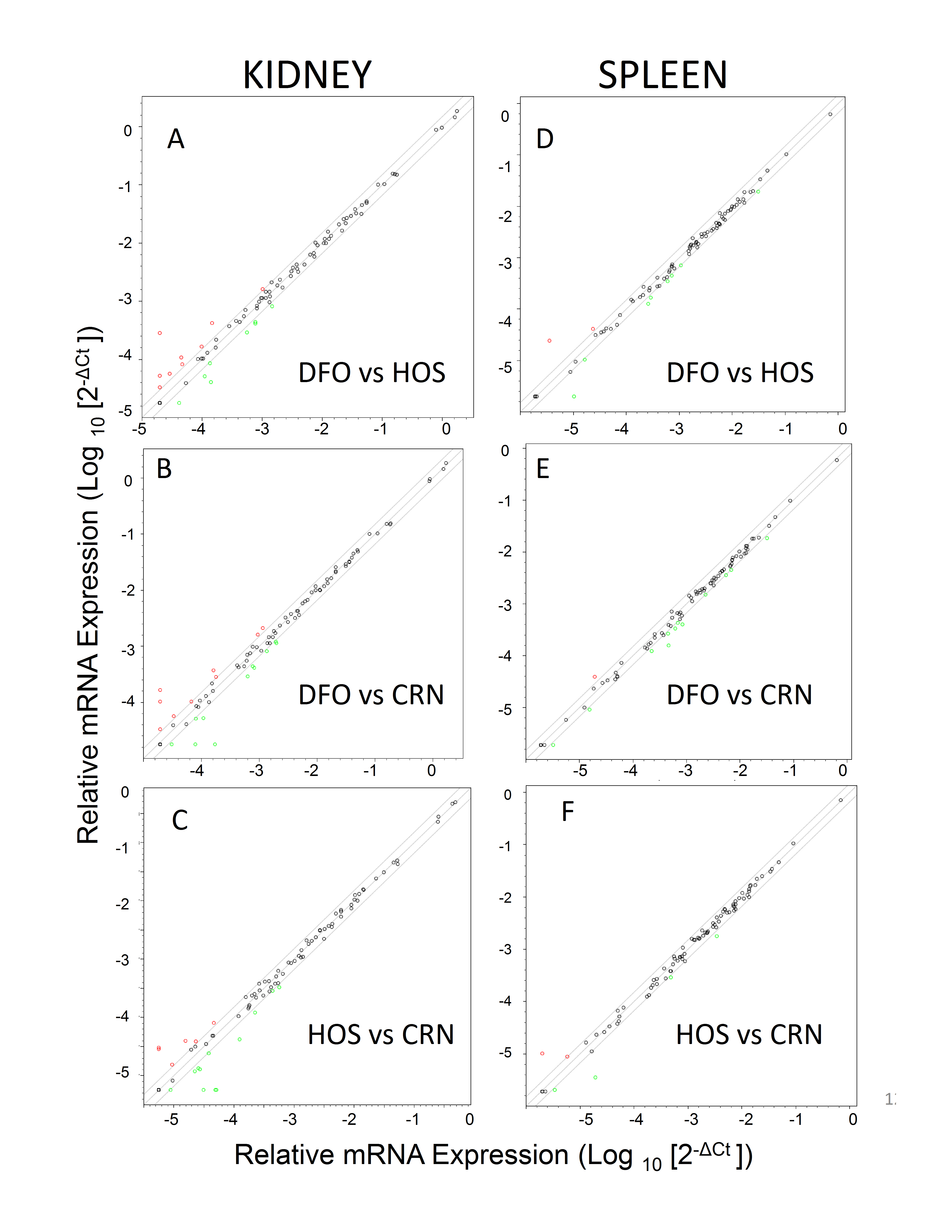

Supplement: Figure S1 — Comparative effects of n-3 PUFA consumption on CD4+ T cell-related gene expression in kidneys and spleens of 16 wk old NZBWF1/J mice. Cohorts of mice were fed CRN, HOS, or DFO diets for 30 wk beginning at 4 wk. At wk 34, mice were euthanized and mRNA isolated from kidneys. Extracted mRNA were pooled and analyzed using a SABioscience Mouse Th1-Th2-Th3 PCR Array. Points outside of solid line are>1.5–fold difference. (TIF) [file pone.0100255.s001.tif]
