# Supplementary material for: Comparative Effects of n-3, n-6 and n-9 Unsaturated Fatty Acid-Rich Diet Consumption on Lupus Nephritis, Autoantibody Production and CD4+ T Cell-Related Gene Responses in the Autoimmune NZBWF1 Mouse
Source: PLoS One. 2014 Jun 19;9(6):e100255. doi: 10.1371/journal.pone.0100255 (PMC4063768; doi:10.1371/journal.pone.0100255)
Supplement: Table S1 — Differential effects of consuming n-3, n-6 and n-9 PUFAs on expression of CD4+ T cell-related genes in kidneys and spleens of 16 wk old female NZBWF1 mice. (DOCX) [file pone.0100255.s002.docx]

| **Table S1. Differential effects of consuming n-3, n-6 and n-9 PUFAs on expression of CD4^+^ T cell-related genes in kidneys and spleens of 16 wk old female NZBWF1 mice^a,b^** | | | | | | | |
| --- | --- | --- | --- | --- | --- | --- | --- |
| **Gene ID** | **Gene Description** | | | **KIDNEY** | | **SPLEEN** | |
|  |  |  |  | **DFO**  **vs CRN** | **DFO**  **vs**  **HOS** | **DFO**  **vs CRN** | **DFO**  **vs**  **HOS** |
| **Integral Membrane Protein** | | | |  |  |  |  |
| Ccr2 | Chemokine (C-C motif) receptor 2 | | | 1.06 | 1.19 | 1.06 | 1.34 |
| Ccr3 | Chemokine (C-C motif) receptor 3 | | | -1.16 | 1.08 | -1.16 | 1.01 |
| Ccr4 | Chemokine (C-C motif) receptor 4 | | | -1.30 | 1.18 | -1.30 | 1.27 |
| Ccr5 | Chemokine (C-C motif) receptor 5 | | | 1.09 | 1.15 | 1.09 | -1.41 |
| Ccr10 | Chemokine (C-C motif) receptor 10 | | | -1.72 | -1.02 | -1.72 | -2.05 |
| Cd4 | CD4 antigen | | | -1.23 | -1.78 | -1.23 | -1.32 |
| Cd27 | CD27 antigen | | | -2.15 | -1.94 | -2.15 | -1.31 |
| Cd28 | CD28 antigen | | | -1.72 | 1.05 | -1.72 | -1.22 |
| Cd40 | CD40 antigen | | | 1.17 | 1.06 | 1.17 | -1.36 |
| Cd40lg | CD40 ligand | | | 1.62 | 14.16 | 1.62 | -1.06 |
| Cd80 | CD80 antigen | | | 8.63 | 1.66 | 8.63 | -1.11 |
| Cd86 | CD86 antigen | | | -1.29 | -1.27 | -1.29 | -1.14 |
| Ctla4 | Cytotoxic T-lymphocyte-associated protein 4 | | | -1.52 | -2.17 | -1.52 | -1.59 |
| Cxcr3 | Chemokine (C-X-C motif) receptor 3 | | | -1.25 | 1.01 | -1.25 | -1.21 |
| Icos | Inducible T cell co-stimulator | | | 1.08 | -1.39 | 1.08 | -1.25 |
| Igsf6 | Immunoglobulin superfamily, member 6 | | | -1.07 | -1.19 | -1.07 | -1.13 |
| Il1r1 | Interleukin 1 receptor, type I | | | 1.04 | -1.29 | 1.04 | 1.07 |
| Il2ra | Interleukin 2 receptor, alpha chain | | | -2.02 | 2.63 | -2.02 | -1.01 |
| Il4ra | Interleukin 4 receptor, alpha | | | 1.09 | -1.11 | 1.09 | -1.03 |
| Il12rb2 | Interleukin 12 receptor, beta 2 | | | -1.70 | ND | -1.70 | -1.75 |
| Il13ra1 | Interleukin 13 receptor, alpha 1 | | | -1.01 | -1.12 | -1.01 | -1.05 |
| Il18r1 | Interleukin 18 receptor 1 | | | 1.15 | 1.33 | 1.15 | 1.04 |
| Il27ra | Interleukin 27 receptor, alpha | | | -1.61 | -1.80 | -1.61 | -1.24 |
| Ptprc | Protein tyrosine phosphatase, receptor type C | | | 1.08 | 1.05 | 1.08 | -1.58 |
| Tlr4 | Toll-like receptor 4 | | | 1.05 | -1.01 | 1.05 | 1.00 |
| Tlr6 | Toll-like receptor 6 | | | 1.88 | 1.46 | 1.88 | -1.01 |
| Tmed1 | Transmembrane emp24 domain containing 1 | | | 1.16 | 1.10 | 1.16 | 1.07 |
| Tnfrsf4 | Tumor necrosis factor receptor superfamily, member 4 | | | -1.18 | 1.16 | -1.18 | -1.30 |
| Tnfrsf8 | Tumor necrosis factor receptor superfamily, member 8 | | | ND | ND | ND | -1.26 |
| **Kinases** | | | | | | | |
| Jak1 | Janus kinase 1 | -1.21 | | | -1.18 | -1.21 | -1.01 |
| Jak2 | Janus kinase 2 | -1.13 | | | 1.04 | -1.13 | 1.27 |
| Jak3 | Janus kinase 3 | 1.27 | | | 1.06 | 1.27 | -1.31 |
| Junb | Jun-B oncogene | -1.09 | | | -1.28 | -1.09 | -1.41 |
| Mapk8 | Mitogen-activated protein kinase 8 | 1.08 | | | 1.25 | 1.08 | -1.09 |
| Mapk9 | Mitogen-activated protein kinase 9 | -1.06 | | | -1.04 | -1.06 | 1.09 |
| Tyk2 | Tyrosine kinase 2 | -1.11 | | | -1.17 | -1.11 | -1.07 |
| **Cytokine/Chemokine** | | | | | | | |
| Ccl5 | Chemokine (C-C motif) ligand 5 | | -1.33 | | -1.06 | -1.33 | -1.44 |
| Ccl7 | Chemokine (C-C motif) ligand 7 | | 1.13 | | 1.24 | 1.13 | 1.05 |
| Ccl11 | Chemokine (C-C motif) ligand 11 | | -9.39 | | ND | -9.39 | -1.01 |
| Csf2 | Colony stimulating factor 2 (granulocyte-macrophage) | | ND | | ND | ND | ND |
| Ifng | Interferon gamma | | 5.37 | | -1.03 | 5.37 | -1.05 |
| Il2 | Interleukin 2 | | -4.31 | | -2.34 | -4.31 | 1.20 |
| Il4 | Interleukin 4 | | 1.08 | | -1.58 | 1.08 | -1.55 |
| Il5 | Interleukin 5 | | -1.33 | | 1.11 | -1.33 | -1.01 |
| Il6 | Interleukin 6 | | 1.73 | | 1.97 | 1.73 | 6.83 |
| Il7 | Interleukin 7 | | -1.08 | | 1.10 | -1.08 | -1.05 |
| Il9 | Interleukin 9 | | 1.03 | | 1.26 | 1.03 | ND |
| Il10 | Interleukin 10 | | 1.16 | | 2.38 | 1.16 | 1.02 |
| Il12b | Interleukin 12B | | 1.46 | | 1.27 | 1.46 | -1.18 |
| Il13 | Interleukin 13 | | 1.74 | | 1.66 | 1.74 | -1.18 |
| Il15 | Interleukin 15 | | -1.11 | | -1.40 | -1.11 | -1.04 |
| IL17a | Interleukin 17A | | ND | | ND | ND | ND |
| Il18 | Interleukin 18 | | -1.13 | | -1.12 | -1.13 | -1.10 |
| Il23a | Interleukin 23, alpha subunit p19 | | 1.55 | | 1.04 | 1.55 | -1.48 |
| Il27 | Interleukin 27 | | 1.12 | | 1.06 | 1.12 | 1.22 |
| Opn | Osteopontin | | 1.12 | | 1.07 | 1.12 | -1.38 |
| Tgfb3 | Transforming growth factor, beta 3 | | 1.31 | | 1.11 | 1.31 | -1.12 |
| Tnf | Tumor necrosis factor | | 1.02 | | -1.07 | 1.02 | -1.09 |
| Tnfsf4 | Tumor necrosis factor superfamily, member 4 | | ND | | ND | ND | 1.08 |
| **Transcription Factors and Regulators** | | | | | | | |
| Bcl6 | B cell leukemia/lymphoma 6 | | 1.26 | | 1.13 | 1.26 | -1.21 |
| Cebpb | CCAAT/enhancer binding protein (C/EBP), beta | | 1.28 | | 1.19 | 1.28 | -1.07 |
| Crebbp | CREB binding protein | | 1.07 | | -1.06 | 1.07 | -1.01 |
| Gata3 | GATA binding protein 3 | | 1.08 | | -1.09 | 1.08 | -1.15 |
| Gfi1 | Growth factor independent 1 | | -1.32 | | -3.44 | -1.32 | -1.22 |
| Irf1 | Interferon regulatory factor 1 | | 1.06 | | -1.13 | 1.06 | -1.19 |
| Irf4 | Interferon regulatory factor 4 | | 2.34 | | 1.32 | 2.34 | -1.17 |
| Maf | V-maf AS42 oncogene homolog | | 1.08 | | 1.04 | 1.08 | -1.18 |
| Nfatc1 | NF of activated T cells, cyto., calcineurin-dep. 1 | | -1.12 | | -1.06 | -1.12 | -1.18 |
| Nfatc2 | NF of activated T cells, cyto., calcineurin-dep. 2 | | -1.00 | | -1.09 | -1.00 | -1.22 |
| Nfatc2ip | Natc2 interacting protein | | -1.03 | | 1.15 | -1.03 | 1.09 |
| Nfatc3 | NF of activated T cells, cyto., calcineurin-dep. 3 | | -1.08 | | -1.14 | -1.08 | -1.01 |
| Nfkb1 | NF of Κ light PP gene enhancer in B cells 1, p105 | | -1.04 | | -1.31 | -1.04 | -1.12 |
| Pcgf2 | Polycomb group ring finger 2 | | 1.21 | | -1.40 | 1.21 | -1.03 |
| Stat1 | Signal transducer and activator of transcription 1 | | -1.04 | | 1.07 | -1.04 | -1.26 |
| Stat4 | Signal transducer and activator of transcription 4 | | ND | | ND | ND | 1.76 |
| Tbx21 | T-box 21 | | -1.07 | | 1.72 | -1.07 | -1.32 |
| Tcfcp2 | Transcription factor CP2 | | -1.08 | | 1.29 | -1.08 | -1.49 |
| Yy1 | YY1 transcription factor | | -1.04 | | -1.06 | -1.04 | 1.08 |
| **Miscellaneous** | | |  | |  |  |  |
| Inha | Inhibin alpha | | -1.56 | | -1.13 | -1.56 | -1.35 |
| Il18bp | Interleukin 18 binding protein | | -1.10 | | -1.11 | -1.10 | -1.05 |
| Socs1 | Suppressor of cytokine signaling 1 | | -1.95 | | -1.86 | -1.95 | -1.52 |
| Socs3 | Suppressor of cytokine signaling 3 | | 1.76 | | 1.57 | 1.76 | -1.73 |
| Socs5 | Suppressor of cytokine signaling 5 | | 1.08 | | -1.03 | 1.08 | -1.05 |
| Sftpd | Surfactant associated protein D | | -1.02 | | 2.86 | -1.02 | -5.16 |

^a^ For qRT-PCR array comparisons, RNA expression values obtained from the kidneys of female NZBWF1/J mice fed docosahexaenoic acid-enriched diets were made relative to specified feeding group values and expressed as fold change. Expression > 1.5 fold change was considered noteworthy.

^b^ Abbreviations: CRN, corn oil-enriched diet; SAF, high-oleic safflower oil-enriched diet; DHA, docosahexaenoic acid ethyl ester-enriched diet; ND, not detected.
